# The disease formerly known as rheumatoid arthritis

**DOI:** 10.1186/ar4593

**Published:** 2014-06-26

**Authors:** Gary S Firestein

**Affiliations:** 1 Division of Rheumatology, Allergy, and Immunology, UC San Diego School of Medicine, 9500 Gilman Drive, La Jolla, CA 92093, USA

## Abstract

Rheumatoid arthritis is a complex disease where predetermined and stochastic factors conspire to confer disease susceptibility. In light of the diverse responses to targeted therapies, rheumatoid arthritis might represent a final common clinical phenotype that reflects many pathogenic pathways. Therefore, it might be appropriate to begin thinking about rheumatoid arthritis as a syndrome rather than a disease. Use of genetics, epigenetics, microbiomics, and other unbiased technologies will probably permit stratification of patients based on mechanisms of disease rather than by clinical phenotype.

## 

Observer la nature, et suivez la route qu’elle vous trace.

JJ Rousseau, quoted in [1].

Over 150 years ago, Garrod coined the term ‘rheumatoid arthritis’ (RA) to distinguish it from other forms of arthritis, most notably gout and acute rheumatism [1]. Years later, disease subsets were further characterized based, in part, on clinical manifestations such as erosions and nodules or laboratory values such as autoantibodies in the blood. For instance, patients with rheumatoid factors and anti-citrullinated protein antibodies (ACPAs) tend to have more severe disease and worse long-term outcomes than seronegative patients.

The broad range of genes associated with RA, the role of the environment in disease initiation, and the diversity of responses to targeted therapies necessitate a re-evaluation of time-honored stratification based on carefully documented clinical phenotypes. Moreover, we should reconsider whether RA should be viewed as the disease that Garrod described or whether it represents a final common pathway of divergent mechanisms in an organ (synovium) with a limited repertoire of responses. In this context, RA could be thought of as a syndrome with multiple etiologic events.

RA susceptibility is determined, in part, by inherited risk factors that are predetermined. The single nucleotide polymorphisms (SNPs) associated with RA are dispersed widely across the genome, with notable concentration in genes that participate in adaptive and innate immune responses [2]. Multiple genome-wide association studies have identified scores of disease-associated SNPs. By far the greatest genetic risk is conferred by the class II major histocompatibility gene HLA-DR, which participates in antigen presentation to T lymphocytes [3]. The critical regions of the encoded protein have been well characterized and are located in and around the antigen-binding groove. However, the observation that identical twins only have perhaps a 15% concordance rate for RA indicates that inherited DNA sequences account for a minority of risk and might not be as important as other influences [4]. Put another way, full diploid genome sequencing of patients ignores over 80% of disease risk.

Many SNPs outside the major histocompatibility complex also contribute to susceptibility, but their influence is much lower, with relative risks typically <1.2 [5]. One need not have all of these SNPs to develop RA; only a limited subset are probably needed in the presence of the proper environmental exposures. Individual and combinations of low-penetrance susceptibility genes have not offered major insights into the clinical phenotype, although it is still early days for these complex analyses. The fact that various combinations of genes and types of environmental stress lead to the same phenotype suggests that we are not looking at a single disease but at a process with multiple pathways.

The “original sin” in ACPA-positive RA is probably due to an interaction between disease-associated HLA-DR genes and the environment, especially at mucosal surfaces (reviewed in [6]). The first steps could be viewed as a normal adaptive immune response against stress-induced modification of peptides, most notably by citrullination. Stochastic events such as smoking, infection, periodontitis, lung inflammation, or the gut microbiome thus lead to induce enzymes (for example, peptidyl arginine deiminases) that alter peptides and produce neo-epitopes not encountered by the thymus during early development. This concept is especially relevant since recent studies suggest that the gastrointestinal flora in early RA might be unique, with an overabundance of *Provatella copri*[7]. These environmental differences could potentially contribute also to altered polarization of T cells to the pathogenic T-helper type 17 phenotype [8].

The autoreactive clones that recognize altered antigens were not deleted during development and can respond appropriately to the antigen. An array of citrullinated peptides fit avidly into the HLA-DR binding groove and activate T cells much more efficiently than the native protein [9]. These early steps probably represent a normal adaptive immune response against altered antigens rather than true autoimmunity. Production of ACPAs directed against a variety of peptides ensues. In the presence of a second hit, such as immune complexes or other mechanisms that activate innate immunity and prepare the synovium, ACPAs gain access to the joint, engage complement, and recruit inflammatory cells that amplify the response. Ultimately, breakdown of tolerance and true autoimmunity against the native proteins ensue, possibly by epitope spread. Interestingly, recently described novel antibody systems to other altered antigens associated with RA, such as through carbamylation rather than citrullination [10], could lead to a similar process.

The most persuasive argument that RA has multiple pathways to the same phenotype is the diversity of responses to highly specific immunotherapies. T-cell co-stimulation blocker, B-cell depletion, tumor necrosis factor inhibitors, or interleukin-6 inhibitors demonstrate similar clinical response rates; that is, about one-half of patients treated with any single agent have a major benefit [11]. If a patient does not respond to one targeted agent, a good response to another agent with a distinct mechanism of action is only slightly less likely than in a biologic-naïve patient [12].

Evaluation of genes or other analytes to stratify patients based on their underlying pathogenesis rather than on clinical phenotype could shed light on how the variable responses occur. Figure 1 shows an example (which is clearly a simplification), focusing only on gene associations. In this model, a patient with clusters of disease-associated SNPs enriched for tumor necrosis factor regulation, for example, might be expected to be a tumor necrosis factor responder. A B-cell genotype, a T-cell genotype, and so on, would also provide clues on how to treat a patient. If no particular clustering occurs and the gene associations are spread across multiple pathways, then any individual targeted therapy would have a low likelihood of success.

**Figure 1:**
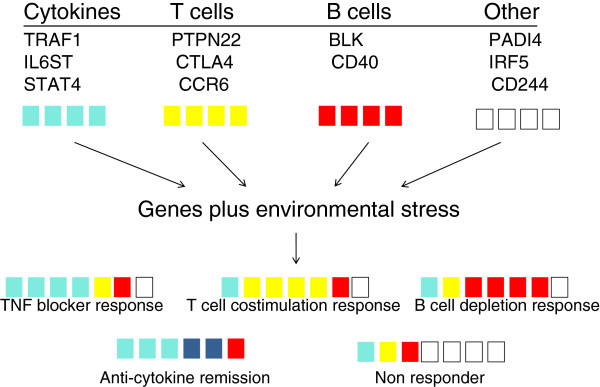
**Simplified schema showing how genes might affect clinical responses to targeted therapies.** Various genes with associated single nucleotide polymorphisms (SNPs) could be generally categorized into various pathogenic mechanisms (for example, tumor necrosis factor (TNF), T cells, B cells, others in this version). A particular individual might only inherit a subset of each of these SNPs. If the majority of inherited SNPs cluster in one mechanism, such as TNF blocker (see bottom rows), then the individual would have a response to the agent that targets this pathway. If the SNPs are not enriched for any particular pathway, then the patient would be a nonresponder. This schema only focuses on SNPs, but would be integrated with pathways that are enriched for epigenetic marks or other regulators of gene expression/function.

As attractive as this notion might be, RA is not that simple and, despite individual studies with potential signals, we cannot reliably predict which patients will respond to a particular biologic despite evaluating many gene associations as well as studies of blood cytokines, synovial pathology, or serum autoantibody profiles. Success will probably require integrating more sophisticated datasets that also take into account many nongenetic influences, such as epigenomics, microbiomics, proteomics, metabolomics, or immunomics, to define the deep profile of a particular individual’s version of RA. Initial studies examining potential pathogenic pathways focusing on DNA methylation in RA synoviocytes or integrating DNA methylation and gene associations in peripheral blood cells provide insights into how this information might begin to identify previously unrecognized subsets [13-15]. Systems biology approaches to nongenetic and genetic influences also permit application of computational methods to test the effects of perturbing networks *in silico*. While this approach is still in its infancy, it could ultimately decrease the need for biologic validation of every potential target or could identify combinations of therapies that will be additive or synergistic.

These observations suggest that RA might be thought of as a collection of distinct mechanisms rather than a single pathway; that is, as a syndrome rather than a disease. A similar conceptual evolution has occurred with other diseases, such as acute myelogenous leukemia, with a transition from phenotype or histologic diagnosis to segmenting the disease by genotype. We face the reverse of past progress in medicine, where a unifying cause ultimately links many clinical phenotypes, such as the great imitator syphilis. Instead, our understanding of RA as a clinical phenotype is devolving into multiple pathogenic pathways. RA might have a common entry point, such as adaptive immune responses to altered peptides followed by immune complexes and autoimmunity, but the subsequent byzantine pathway to the clinical phenotype is so convoluted and personalized that solving RA for a particular patient requires a systems approach using multiple emerging technologies.

We have come a long way from “acute rheumatism”, but still have far to go before these pathogenic processes can be meaningfully dissected. The therapeutic successes with the average patient have been stunning, but we have reached the limit of this traditional approach. We must begin the process of deconvoluting RA using unbiased technology and carefully integrating predetermined and stochastic influences that lead to the syndrome we call RA.

## Note

This article is part of the collection ‘*Why is there persistent disease despite aggressive therapy of rheumatoid arthritis?*’, edited by Pierre Miossec. Other articles in this series can be found at http://arthritis-research.com/series/residual.

## Abbreviations

ACPA: Anti-citrullinated protein antibody; RA: Rheumatoid arthritis; SNP: Single nucleotide polymorphism.

## Competing interests

The author declares that he has no competing interests.
